# Is Increased Biofilm Formation Associated with Decreased Antimicrobial Susceptibility? A Systematic Literature Review

**DOI:** 10.3390/microorganisms13102292

**Published:** 2025-10-01

**Authors:** Abhinav Madduri, Lobke Vanommeslaeghe, Tom Coenye

**Affiliations:** Laboratory of Pharmaceutical Microbiology, Ghent University, 9000 Ghent, Belgium; abhinavm955@gmail.com (A.M.);

**Keywords:** biofilm, antimicrobial susceptibility, antimicrobial resistance

## Abstract

Biofilm formation is a key factor in microbial survival and persistence, often contributing to reduced antimicrobial susceptibility. This systematic literature review investigates whether increased biofilm formation correlates with decreased antibiotic susceptibility. The literature search was conducted in the Pubmed database and we identified and screened 328 studies, with 35 ultimately meeting the inclusion criteria for detailed analysis. Findings reveal that the relationship between biofilm size and antimicrobial susceptibility is highly variable and influenced by multiple factors, including microbial species, strain-specific traits, antibiotic type, and experimental methodologies. While some studies report a positive correlation between biofilm biomass and reduced susceptibility, others show weak or no such relationships, and statistical support for a correlation is often lacking (also due to small sample sizes). The lack of standardized biofilm quantification methods and susceptibility metrics further complicates cross-study comparisons. These findings underscore the need for standardized protocols and more comprehensive datasets to clarify the complex interplay between biofilm formation and antibiotic susceptibility. Regardless of these difficulties, the available data clearly indicate that ‘bigger’ biofilms are not by definition less susceptible. Future research should prioritize diverse and sufficiently large strain collections and consistent methodologies to better understand and address biofilm-associated antimicrobial tolerance.

## 1. Introduction

The majority of chronic and/or medical device-related infections in humans are biofilm-associated. Biofilms are aggregates of surface-associated or tissue-embedded microbial cells that are enclosed within an extracellular polymeric substance matrix [1,2]. One of the hallmarks of biofilm-related infections is the difficulty of successful antimicrobial treatment, as biofilm cells frequently exhibit reduced susceptibility [3,4,5]. There are several factors involved in the reduced susceptibility of biofilms to antibiotics, including the blocking and repelling of antibiotics by the extracellular matrix [2,3]. An example of this is the reduced penetration of the glycopeptide antibiotic vancomycin into *Staphylococcus epidermidis* biofilms, which is mediated by an increase in the extracellular DNA (eDNA) concentration [6]. Similarly, binding of the negatively charged aminoglycoside antibiotic tobramycin to alginate (an important polysaccharide component in the *Pseudomonas aeruginosa* biofilm matrix) can reduce tobramycin concentrations in deeper layer of the biofilm [7]. In addition, the accumulation of antibiotic-degrading enzymes in the matrix (e.g., β-lactamase in *P. aeruginosa* biofilms [8]) could contribute to reduced biofilm susceptibility. Other factors involved in reduced susceptibility of biofilms include changes in metabolism that lead to reduced production of antibiotic-induced reactive oxygen species as well as the presence of biofilm-specific efflux pumps [5,9,10,11,12]. In addition, the structural and metabolic heterogeneity often observed in biofilms leads to gradients (e.g., of oxygen and nutrients) that can affect physiology of organisms (e.g., by inducing a state of dormancy) and affect antimicrobial susceptibility [13,14].

While many studies have investigated mechanisms of biofilm formation and how biofilms respond to exposure to antibiotics under different conditions, it remains unclear whether there is a correlation between the amount of biofilm formed (i.e., biofilm ‘size’ or ‘thickness’) and antimicrobial susceptibility. While this may seem like a simple question at first sight, a combination of several factors determines the biofilm phenotype, including the species involved and the environmental conditions under which the biofilm is formed and/or exposed to antibiotics [15,16,17,18]. In addition, biofilms are quantified using a wide range of approaches and it is important to keep in mind that these different approaches frequently measure/quantify different aspects of biofilms and each come with their own advantages and limitations [19]. For example, the frequently used crystal violet and resazurin-based staining approaches allow quantification of total biomass (live and dead cells as well as some matrix components) and the number of metabolically active cells, respectively, and results obtained with these different approaches may yield very different outcomes. Moreover, results obtained with these indirect quantification approaches based on various chemical stainings may yield results that are not necessarily in line with those obtained by determining the number of culturable cells (i.e., determination of the number of colony forming units [CFU]) [19,20].

In the present study, we report on a systematic literature review that was conducted to investigate whether the amount of biofilm formed correlates with antibiotic susceptibility, or phrased differently: are biofilms with greater biomass or thickness less susceptible to antibiotics?

## 2. Materials and Methods

### 2.1. Search Strategy

This systematic review was conducted following the PRISMA (Preferred Reporting Items for Systematic Reviews and Meta-Analyses) guidelines [21]. Publications were retrieved from the PubMed electronic database (https://pubmed.ncbi.nlm.nih.gov/) using free text words. The search was restricted to articles published up to 31 July 2025. The final input for the search was ‘biofilm thickness OR biofilm quantification OR biofilm biomass AND antibiotic susceptibility’ to maximize the number of publications for subsequent screening. This review was not registered and a protocol was not prepared.

### 2.2. Study Selection and Exclusion Criteria

**Figure 1:**
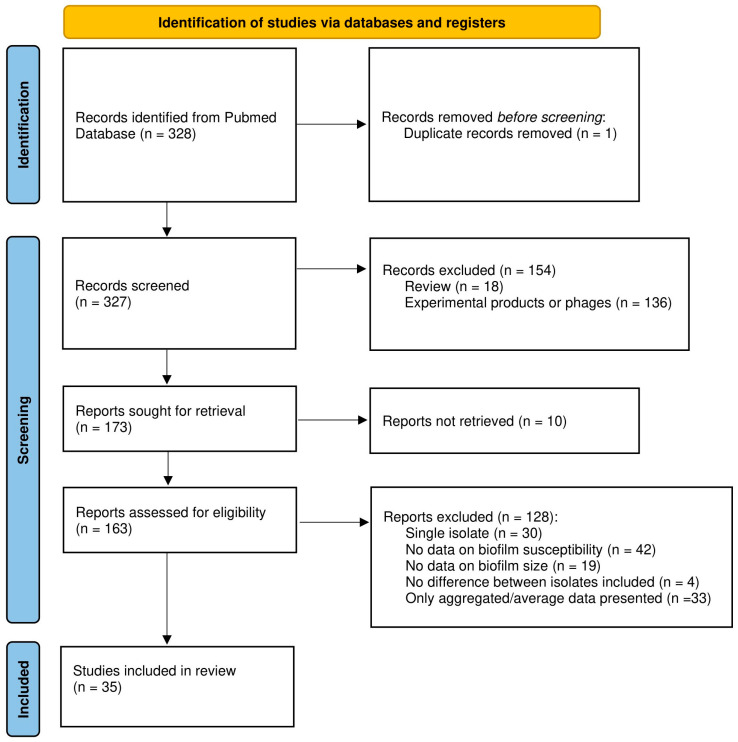
PRISMA flowchart of the study selection process.

Abstracts and full texts from articles were screened by two independent reviewers (A.M. and T.C., or L.V. and T.C.) to collect data on organism(s), biofilm model system(s), quantification method(s), antimicrobial compound(s), and data on biofilm size and susceptibility. Reviews, publications for which we could not access the full text at the time of the search, and studies using phages or experimental products instead of conventional antibiotics were excluded. As it was our goal to compare data obtained with biofilms of different sizes, studies in which only a single isolate and/or a single time point was included, studies in which no relevant data on (biofilm) susceptibility and/or biofilm size were reported (e.g., studies reporting only the minimum inhibitory concentration (MIC) and/or minimum bactericidal concentration (MBC) data were excluded as these values do not accurately reflect biofilm susceptibility [4]), and studies lacking data for the individual strains tested (i.e., studies in which only average or otherwise aggregated data were presented) were excluded. Finally, for several studies data reported indicate that the isolates included had virtually identical biofilm biomass and susceptibility. As answering the question whether increased biofilm biomass correlates with decreased antibiotic susceptibility requires isolates with different biomass and/or different susceptibility, these studies were also excluded. To avoid bias, no other selection criteria were used. Due to lack of formal criteria to assess the quality of non-clinical biofilm studies and the lack of widely accepted guidelines for reporting on such studies, these criteria were not used for selecting studies (i.e., quality of, and potential bias in, the different studies were not assessed and were not used as selection criteria). The study selection process is illustrated in the PRISMA flowchart (Figure 1).

### 2.3. Statistical Analysis

When raw data were included in the main text and/or supplementary data of the studies retrieved, these were used for statistical analysis. However, as many publications retrieved in the framework of this systematic review did not include raw data, we frequently had to estimate data on susceptibility and/or biofilm biomass from figures provided in the publications (e.g., by measuring the height of bars in bar charts). Goodness of fit between two parameters was assessed by calculating the Pearson correlation coefficient (r^2^) as well as the significance of the correlation. The latter allowed to determine whether the slope of the linear regression curve significantly differed from zero based on a two-tailed *t*-test (*p*-values < 0.05 were considered significant). Statistical analyses were carried out using GraphPad Prism (v10.5.0).

## 3. Results

### 3.1. Results of the Literature Search

From the 328 studies initially identified, 293 were excluded for various reasons or not retrieved, leaving 35 papers that were analyzed in detail, as outlined in the PRISMA flowchart (Figure 1). Most studies identified during the initial screening were removed because they pertained to experimental products or phages (*n* = 136), because no data on biofilm susceptibility were included (*n =* 42), or because only average or otherwise aggregated data were presented (*n =* 33). The complete list of the publications analyzed, along with the number of strains included, biofilm model system(s), biofilm quantification method, and antibiotic(s) tested is presented in Table A1 (note that some studies include data on multiple species and are listed several times).

### 3.2. General Overview of Results

The diversity in terms of species investigated, model systems and methods for biofilm quantification used, number of strains included, and/or antibiotics tested makes it difficult to make in-depth and/or across the board comparisons between studies; however, a selection of publications is discussed in more detail below. The studies that included appropriate (raw) data on a sufficient number of strains to allow for the calculation of r^2^ values between two relevant parameters are listed in Table 1. While the majority of these studies pertain to *Staphylococcus aureus*, the table also includes studies with *Candida albicans*, *Candida glabrata*, *Candida krusei*, and *Helicobacter pylori*. Out of the 35 studies analyzed for this review, only one study had this systematic review’s topic as its primary focus [22]. Overall, a wide range of r^2^ values (and corresponding *p*-values) is found based on the data reported in different studies. There are a number of factors potentially influencing the antimicrobial susceptibility of microbial biofilms of different sizes. These are discussed below and illustrated with representative examples.

### 3.3. Species- and Strain-Dependent Differences

A first hypothesis that we addressed in this systematic review is that the relationship between biofilm size and susceptibility is species-dependent (i.e., there is a relationship between size and susceptibility in species A, but no—or an inverse—correlation is found in species B). Example of studies that supports this are the study by Oschmann-Kadenbach et al. [31] and Silva et al. [23]. In these studies a strong correlation was found between biofilm size and susceptibility towards amikacin for *Mycobacteroides abscessus* [31], while low r^2^ values were found for *S. aureus* [23] (Table 1) (it should, however, be noted that statistical analysis indicated this correlation in both cases was not significant; this is discussed further down). A second example is amphotericin B: while a low r^2^ value is observed for *C. albicans*, a high value is reported for *C. krusei* (Table 1) [28]. These results seem to suggest that species-specific differences indeed may occur.

**Figure 2:**
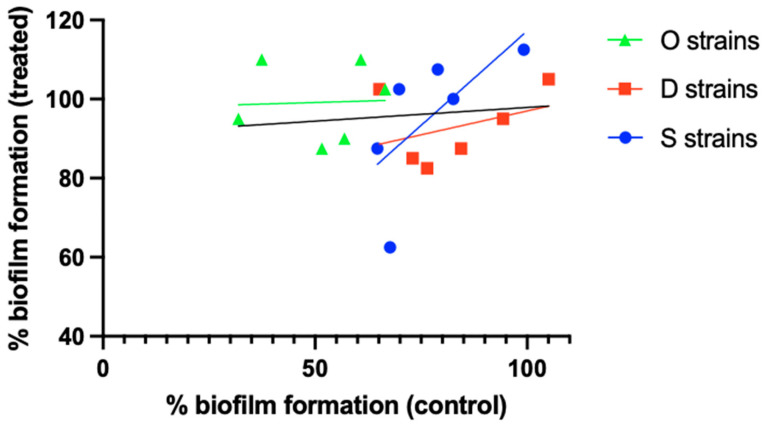
Differences in the relationship between biofilm formation and biofilm ciprofloxacin susceptibility between different *S. aureus* strain sets. O strains (green), D strains (red), and S strains (blue) were isolated from cases of osteomyelitis, diabetic foot ulcers, or bacteremia, respectively. Individual data points and linear correlation curves per subset are shown. The black line indicates the correlation curve for the entire strain collection. Based on the data reported by Silva et al. [24]. Statistical analysis of these data is presented in Table 2.

A second hypothesis is that finding a correlation between biofilm size and susceptibility is strain-dependent (i.e., there is a relationship between size and susceptibility in a particular strain or set of strains of a certain species, but no—or an inverse—correlation is found in a different set of strains belonging to the same species). The results obtained by Silva et al. [24] illustrate this. When comparing relative biofilm formation (i.e., crystal violet absorbance relative to that of *S. aureus* ATCC 25923) in the absence of antibiotic to that after exposure to 4.5 µg/mL ciprofloxacin, a low r^2^ value (0.011) was observed for the overall set of 18 *S. aureus* (MRSA) isolates. However, when these 18 isolates were divided into 3 groups based on their isolation source (osteomyelitis [O strains], diabetic foot [D strains], or bacteremia [S strains]), a different picture emerged (Figure 2), with r^2^ values of 0.002, 0.142, and 0.450 for the O, D, and S strains, respectively. Although these results suggest that the relationship between biofilm formation and antibiotic susceptibility can depend on the (sub)set of *S. aureus* isolates studied, statistical analysis of the full dataset as well as the subsets revealed that none of the correlations is significant, despite considerable differences in *p*-values (Table 2).

### 3.4. Antibiotic-Dependent Differences

**Figure 3:**
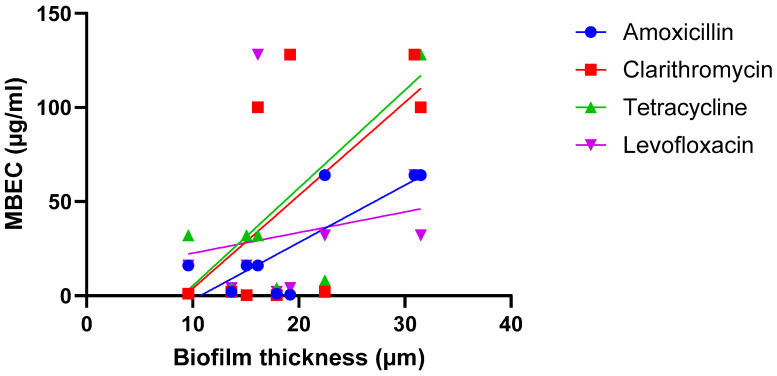
Exposure of nine *H. pylori* isolates to four antibiotics suggests the relationship between biofilm formation (quantified using microscopy) and biofilm susceptibility (as quantified by the MBEC) is antibiotic dependent. Data were initially reported by Wu et al. [22]. Statistical analysis of these data is presented in Table 2.

A third hypothesis is that the correlation between biofilm size and susceptibility is antibiotic-dependent (i.e., there is a relationship between size and susceptibility towards antibiotic A, but no—or an inverse—correlation is found for antibiotic B) and several studies point in the direction of this hypothesis being true. In the work conducted by Wu et al. on *H. pylori*, strong and significant correlations were found when biofilms were exposed to amoxicillin or tetracycline, while weaker and non-significant correlations were found after biofilms were exposed to clarithromycin or levofloxacin [22] (Figure 3; Table 2).

**Figure 4:**
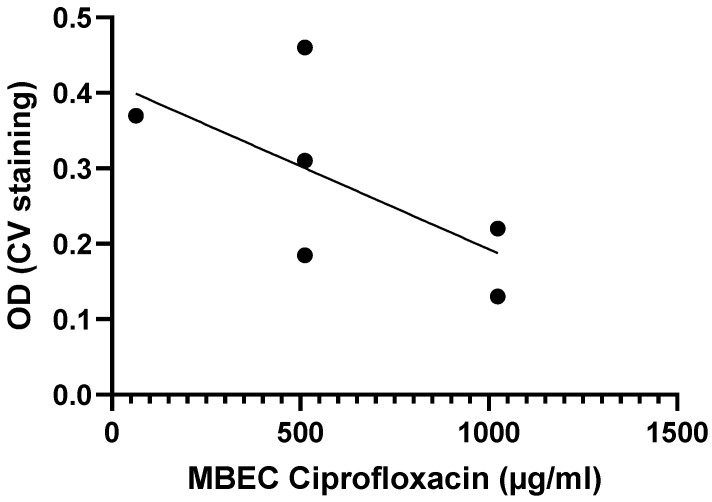
Exposure of six *B. cenocepacia* isolates to ciprofloxacin suggests there is a moderate, inverse correlation between biofilm formation (quantified using crystal violet staining) and biofilm susceptibility (as quantified by the MBEC) to ciprofloxacin. Data were initially reported by Tomlin et al. [29]. Statistical analysis of these data is presented in Table 2.

Another example is from the work done by Tomlin et al., who exposed biofilms from six *Burkholderia cenocepacia* isolates to varying concentrations of ciprofloxacin, ceftazidime, chloramphenicol, and meropenem for 24 h to determine MBEC values [29]. While the MBEC values for ceftazidime, meropenem, and chloramphenicol were above 1024 µg/mL for all six strains, the MBEC values for ciprofloxacin tended to be higher for strains with lower biomass, suggesting a possible inverse correlation between biofilm formation and antibiotic susceptibility for *B. cenocepacia* (Figure 4), although this correlation is not significant (Table 2).

These differences can also be found in the data reported by Alves and colleagues, who collected data on biofilm susceptibility (towards four antifungal drugs) for four different *Candida* species [28]. For the species *C. krusei*, high (and significant) r^2^ values were found for the antifungal drugs fluconazole and amphotericin B, while more moderate (and non-significant) correlations were found for voriconazole and anidulafungin (Table 1) [28]. For *C. albicans*, on the other hand, low (non-significant) correlations between biofilm formation and fluconazole and amphotericin B susceptibility was found, while this correlation was moderate (albeit still not significant) for voriconazole and anidulafungin (Table 1) [28]. Combined, these data suggest that the relationship between biofilm formation and antimicrobial susceptibility can indeed depend on the antimicrobial agent investigated.

### 3.5. Impact of the Model System, Quantification Approach, and Other Experimental Parameters

**Figure 5:**
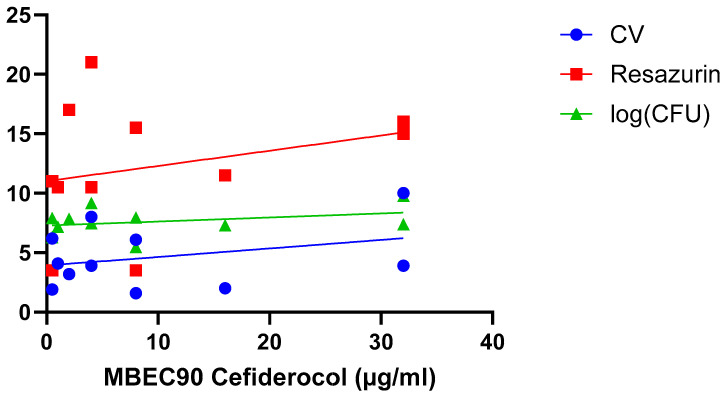
Exposure of 11 *P. aeruginosa* isolates to cefiderocol indicates there is no correlation between biofilm formation (quantified using crystal violet staining [blue], resazurin viability staining [red], and CFU counts [green]) and biofilm susceptibility (as quantified by the MBEC_90_) to cefiderocol. Data were initially reported by Fabrizio et al. [30]. Statistical analysis of these data is presented in Table 2.

A final hypothesis is that the correlation between biofilm susceptibility and size depends on the experimental parameters used in a particular study, including biofilm model system, biofilm age, treatment time, quantification approach, etc. This has been addressed in two studies. Wu et al. investigated differences in susceptibility towards linezolid between 6 h old and 24 h old *S. aureus* biofilms, using both crystal violet staining (which allows to quantify ‘total biomass’, as it binds to both dead and living cells, as well as some matrix components) and resazurin-based viability staining (which allows to quantify the number of metabolically active cells) [26]. For both stainings a strong and significant correlation was found for 6 h old biofilm, while for the 24 h old biofilms only a moderate (but non-significant) correlation was observed for crystal violet (Table 2) (i.e., this study suggests both approaches reveal the same trend). Fabrizio et al. compared susceptibility of *P. aeruginosa* biofilm to cefiderocol using crystal violet and resazurin-based viability staining as well as determination of the number of CFU (which allows to quantify the number of culturable cells) [30] (Figure 5; Table 2). In this particular study, none of the quantification approaches used found a significant correlation between biofilm size and cefiderocol susceptibility (i.e., also data from this study suggest the different approaches yield the same outcome).

## 4. Discussion

As outlined above, the answer to the question ‘*Is increased biofilm formation associated with decreased antimicrobial susceptibility?*’ likely depends on the species, the strain collection, the antimicrobial agent investigated, and/or the experimental setup. Moreover, it seems plausible that a combination of several (or even all) of these factors play an important role.

While there are studies that found at least a moderately positive correlation between the amount of biofilm formed and reduced antimicrobial susceptibility, this is not always the case, and statistical support for this correlation is often lacking. The latter can at least partially be attributed to small sample sizes (most studies retrieved contain data for less than 10 isolates, and only very few contain data for more than 20 isolates; Table A1). As a consequence, overemphasizing the relevance of such observations and/or extending these observations to other species/antibiotic combinations than those investigated seems ill advised.

In addition, careful analysis of the existing literature identified a number of important points of attention for future studies.

First of all, the strain-dependent effects observed in several studies indicate that studies investigating the link between biofilm formation and antimicrobial susceptibility should be based on sufficiently large and diverse strain collections. An added advantage of larger strain collections is the ability to carry out properly powered statistical analyses. We illustrate this point by a closer look at the data reported by Alves et al. [28] for three *Candida* species (*C. albicans*, *C. glabrata*, and *C. krusei*) (Table 1 and Table 2). As mentioned above, when data are analyzed for each species separately, only a minority of the correlations observed are significant. However, when these data are pooled (and data for two *C. valida* strains are added; this increases the number of datapoints to 24), a different picture emerges, with all correlations being significant (Table 1 and Table 2).

Secondly, it is well known that biofilm susceptibility depends on the test conditions [4,32,33]. While various model systems were used across the studies analyzed for this systematic review (Table A1), we did not identify studies in which this question was addressed in a systematic way in multiple model systems for the same collection of strains. Because of this, we cannot definitively answer the question whether the relation between biofilm formation and biofilm susceptibility is model system-dependent, but it seems reasonable to speculate that it is. Considerable efforts have been made to develop standardized and reproducible biofilm approaches (including several ASTM standard test methods) [34,35] as well as clinically relevant ‘in vivo-like’ models [36,37,38] and the further use of these models might help to address the question about the relationship between biofilm size and susceptibility in a more systematic way.

Thirdly, a large variety of biofilm quantification approaches is used and these measure very different aspects of biofilm biology and chemistry [19,20,39]. This lack of standardization when it comes to quantification makes it difficult to compare studies and to extrapolate data. The dataset compiled did not allow us to address the question whether certain quantification approaches would be better than others, but it is important to emphasize that a thorough validation of the quantification method used (for the organism and antimicrobial agent being investigated) is essential. Crucial aspects of such validation include repeatability (i.e., the within lab variation), reproducibility (i.e., the between lab variation), and responsiveness (i.e., the ability of a method to differentiate between the effect of different concentrations of an antibiotic) [39].

In addition, different studies often use different biofilm susceptibility parameters, further complicating comparisons between studies, and a considerable number of papers were excluded from this systematic review, as they erroneously use MIC values as a proxy for biofilm susceptibility [4]. Biofilm-specific susceptibility parameters, including the biofilm prevention concentration (BPC), the minimum biofilm inhibitory concentration (MBIC), and the minimum biofilm eradication concentration (MBEC) have been defined [4,40] and should be used to quantify biofilm susceptibility.

We also noticed that many of the analyzed publications lacked raw data and/or only contained average or otherwise aggregated data. This precludes the re-use of data and analyses like the one performed in the present systematic review. We urge the biofilm community to make raw data available, by including them as part of the publication and/or by depositing them in general or specialized databases. Attempts to create such databases [41] and guidance on a minimal set of metadata to include [42] were made in the past, but have unfortunately remained rather unsuccessful so far.

Finally, a major limitation of the current systematic review is that it had to rely on data from studies that were mostly set up to address other (biological) questions, i.e., very few studies actually specifically investigated whether the amount of biofilm formed correlates with antibiotic susceptibility. It cannot be ruled out that results from studies specifically designed to answer this question yield a different picture than the one painted here. Obviously, this should not be seen as criticism of the studies we discussed in this systematic review but as a suggestion to help move the field forward.

## Figures and Tables

**Table 1:** Summary of results obtained in studies in which r^2^ values between two relevant parameters were calculated. Specific concentrations of antibiotics and/or durations of exposure are only mentioned if, in a study, the same antibiotic was used at different concentrations or for different durations. Abbreviations: CV, crystal violet; CFU, colony forming units; MBEC, minimal biofilm eradication concentration; MIC, minimal inhibitory concentration. * indicates correlations that are statistically significant (*p* < 0.05).

Reference	Organism (No. of Isolates)	Biofilm Quantification	Parameter 1	Parameter 2	Antibiotic and/or Concentration	r^2^ Between Parameters 1 and 2
Silva et al. [23]	*S. aureus* (*n =* 23)	CV Staining	Relative biofilm formation in the absence of antibiotic (compared to reference strain)	Relative biofilm formation in the presence of antibiotic at 10xMIC (compared to the biomass of a biofilm formed in the absence of antibiotic)	Tetracycline	0.009
					Amikacin	0.150
Silva et al. [24]	*S. aureus* (*n =* 18)	CV Staining	Relative biofilm formation in the absence of antibiotic (compared to reference strain)	Relative biofilm formation in the presence of antibiotic (compared to the biomass of a biofilm formed in the absence of antibiotic)	Erythromycin	0.167
					Ciprofloxacin	0.011
					Tetracycline	0.032
Kwiatkowski et al. [25]	*S. aureus* (*n =* 9)	CV Staining	Biofilm biomass in the absence of mupirocin	Biofilm biomass in the presence of mupirocin	Mupirocin	0.253
Wu et al. [26]	*S. aureus* (*n =* 6)	CV and Resazurin Viability Staining	Biofilm biomass in the absence of linezolid	Biofilm biomass in the presence of linezolid (6 h and 24 h; concentration evaluated: MIC)	Linezolid (6 h):	
					CV	0.792 *
					Resazurin	0.773 *
					Linezolid (24 h):	
					CV	0.229
					Resazurin	0.097
Lavoie et al. [27]	*S. aureus* (*n =* 11)	CV Staining	Biofilm biomass in the absence of treatment	MBEC (µg/mL)	Daptomycin	0.137
Alves et al. [28]	*C. albicans* (*n =* 11)	CV Staining	Biofilm biomass in the absence of antifungal compound	Reduction in biofilm biomass following incubation with antifungals (compared to biofilm biomass in the absence)	Fluconazole	0.083
					Voriconazole	0.344
					Anidulafungin	0.362
					Amphotericin B	0.069
Alves et al. [28]	*C. glabrata* (*n =* 3)	CV Staining	Biofilm biomass in the absence of antifungal compound	Reduction in biofilm biomass following incubation with antifungals (compared to biofilm biomass in the absence)	Fluconazole Voriconazole Anidulafungin Amphotericin B	1.101 0.045 0.612 0.039
Alves et al. [28]	*C. krusei* (*n =* 8)	CV Staining	Biofilm biomass in the absence of antifungal compound	Reduction in biofilm biomass following incubation with antifungals (compared to biofilm biomass in the absence)	Fluconazole Voriconazole Anidulafungin Amphotericin B	0.757* 0.462 0.348 0.757*
Alves et al. [28]	All Isolates (*n =* 24) *(C. albicans*, *n =* 11; *C. glabrata*, *n =* 3; *C. krusei*, *n =* 8; *C. valida*, *n =* 2)	CV Staining	Biofilm biomass in the absence of antifungal compound	Reduction in biofilm biomass following incubation with antifungals (compared to biofilm biomass in the absence)	Fluconazole Voriconazole Anidulafungin Amphotericin B	0.410* 0.193* 0.190* 0.302*
Wu et al. [22]	*H. pylori* (*n =* 9)	Microscopy and Determination of the Number of CFU	Biofilm thickness (µm)	MBEC (µg/mL)	Amoxicillin	0.642 *
					Tetracycline	0.485 *
					Clarithromycin	0.377
					Levofloxacin	0.041
Tomlin et al. [29]	*B. cenocepacia* (*n =* 6)	CV Staining	Biofilm biomass in the absence of ciprofloxacin	MBEC (µg/mL)	Ciprofloxacin	0.424
Fabrizio et al. [30]	*P. aeruginosa* (*n =* 11)	CV and Resazurin Viability Staining; Determination of Number of CFU	Biofilm biomass in the absence of treatment	MBEC (µg/mL)	Cefiderocol	
					CV	0.101
					Resazurin	0.079
					CFU	0.114

**Table 2:** Summary of statistical analysis of linear regression performed for selected studies. * indicates correlations that are statistically significant (*p* < 0.05).

Isolate Collection	r^2^	*p*-Value
*S. aureus* [23] (*n =* 23) (amikacin)	0.150	0.068
All *S. aureus* isolates included in Silva et al. [24] (*n =* 18) (ciprofloxacin)	0.011	0.675
*S. aureus* strains isolated from cases of osteomyelitis [24] (*n =* 6) (ciprofloxacin)	0.002	0.935
*S. aureus* strains isolated from cases diabetic foot ulcers [24] (*n =* 6) (ciprofloxacin)	0.142	0.462
*S. aureus* strains isolated from cases of bacteremia [24] (*n =* 6) (ciprofloxacin)	0.450	0.145
*S. aureus* [26] (*n =* 6) (linezolid)		
CV, 6 h old biofilm	0.792	0.018 *
CV, 24 h old biofilm	0.229	0.337
Resazurin, 6 h old biofilm	0.773	0.021 *
Resazurin, 24 h old biofilm	0.097	0.546
*S. aureus* [27] (*n =* 11) (daptomycin)	0.137	0.262
*H. pylori* [22] (*n =* 9)		
Amoxicillin	0.642	0.009 *
Clarithromycin	0.377	0.079
Tetracycline	0.485	0.037 *
Levofloxacin	0.041	0.603
*B. cenocepacia* [29] (*n =* 6) (ciprofloxacin)	0.424	0.161
*M. abscessus* [31] (*n =* 4) (amikacin)	0.775	0.119
*P. aeruginosa* [30] (*n =* 11) (cefiderocol)		
CV	0.101	0.342
Resazurin	0.079	0.403
Log CFU	0.114	0.310
*C. albicans* [28] (*n =* 11)		
Fluconazole	0.083	0.391
Voriconazole	0.344	0.058
Anidulafungin	0.362	0.050
Amphotericin B	0.069	0.463
*C. krusei* [28] (*n =* 8)		
Fluconazole	0.757	0.005 *
Voriconazole	0.462	0.064
Anidulafungin	0.348	0.124
Amphotericin B	0.757	0.005 *
*Candida* spp. [28] (*n =* 24)		
Fluconazole	0.410	<0.001 *
Voriconazole	0.193	0.031 *
Anidulafungin	0.190	0.033 *
Amphotericin B	0.302	0.005 *

## Data Availability

No new data were created or analyzed in this study.

## Abbreviations

The following abbreviations are used in this manuscript:

BPC	Biofilm prevention concentration
CFU	Colony forming unit
CV	Crystal violet
MBEC	Minimum biofilm eradication concentration
MIC	Minimum inhibitory concentration

## Appendix A

**Table A1:** Complete list and summaries of the publications analyzed for the systematic literature review. Studies are grouped per species/genus. Some studies include data on multiple species.

*Staphylococcus aureus*				
Reference	Number of Isolates Included	Model System	Biofilm Quantification Method	Antibiotics Tested
Hon et al. [43]	5	Microtiter plate	Crystal violet staining	Mupirocin
Liang et al. [44]	2	Microtiter plate	Crystal violet staining	Penicillin, ampicillin, meropenem, streptomycin, kanamycin, and gentamicin
Silva et al. [23]	23	Microtiter plate	Crystal violet staining and XTT viability staining	Amikacin and tetracycline
Silva et al. [24]	18	Microtiter plate	Crystal violet staining	Ciprofloxacin, erythromycin, and tetracycline
Kwiatkowski et al. [25]	8	Microtiter plate	Crystal violet staining	Mupirocin
Wu et al. [26]	6	Microtiter plate	Crystal violet staining and resazurin viability staining	Linezolid
de Matos et al. [45]	6	Microtiter plate	Crystal violet staining	Gentamicin, linezolid, rifampicin, and vancomycin
Abdelhady et al. [46]	10	Microtiter plate	Safranin staining	Vancomycin
Wells et al. [47]	2	Microtiter plate	Crystal violet staining	Oxacillin, vancomycin, and ampicillin
Roveta et al. [48]	3	Microtiter plate	Crystal violet staining	Moxifloxacin
Xu et al. [49]	6	Microtiter plate	Crystal violet staining	Daptomycin
Lavoie et al. [27]	11	Microtiter plate	Crystal violet staining	Daptomycin
**Coagulase-Negative *Staphylococci* spp.**				
**Reference**	**Number of Isolates Included**	**Model System**	**Biofilm Quantification Method**	**Antibiotics Tested**
Silva et al. [50]	19	Microtiter plate	Crystal violet staining	Tetracycline and amikacin
***Pseudomonas aeruginosa***				
**Reference**	**Number of Isolates Included**	**Model System**	**Biofilm Quantification Method**	**Antibiotics Tested**
Papa-Ezdra et al. [51]	2	Microtiter plate	Crystal violet staining	Rifampicin, meropenem, gentamicin, amikacin, and ciprofloxacin
Ruhal et al. [52]	3	Glass test tubes	Crystal violet staining	Tobramycin, colistin, and ciprofloxacin
Fortes et al. [53]	2	Microtiter plate	Crystal violet staining	Polymyxin B
Goodyear et al. [54]	10	Microtiter plate	Crystal violet staining	Piperacillin, aztreonam, imipenem, colistin, tobramycin, and ciprofloxacin
Žiemytė et al. [55]	3	Microtiter plate	Crystal violet staining	Ceftazidime, ciprofloxacin, colistin, tobramycin, imipenem, meropenem, and piperacillin-tazobactam
Gupta et al. [56]	4	Flow tube reactor	Crystal violet staining	Tobramycin and norfloxacin
Fabrizio et al. [30]	11	Microtiter plate	Crystal violet staining, resazurin viability staining, and determination of number of CFU	Cefiderocol
***Streptococcus* spp.**				
**Reference**	**Number of Isolates Included**	**Model System**	**Biofilm Quantification Method**	**Antibiotics Tested**
Silva et al. [50]	2	Microtiter plate	Crystal violet staining	Tetracycline and amikacin
Alvim et al. [57]	4	Microtiter plate	Crystal violet staining	Penicillin
Chen et al. [58]	2	Microtiter plate	Crystal violet staining	Chlorhexidine
Hall-Stoodley et al. [59]	6	Microtiter plate	Crystal violet staining	Azithromycin
Roveta et al. [48]	3	Microtiter plate	Crystal violet staining	Moxifloxacin
***Escherichia coli***				
**Reference**	**Number of Isolates Included**	**Model System**	**Biofilm Quantification Method**	**Antibiotics Tested**
Roveta et al. [48]	3	Microtiter plate	Crystal violet staining	Moxifloxacin
***Burkholderia* spp.**				
**Reference**	**Number of Isolates Included**	**Model System**	**Biofilm Quantification Method**	**Antibiotics Tested**
Anutrakunchai et al. [60]	10	Microtiter plate	Crystal violet staining	Ceftazidime
Tomlin et al. [29]	6	Microtiter plate	Crystal violet staining	Ceftazidime, chloramphenicol, ciprofloxacin, and meropenem
***Haemophilus influenzae***				
**Reference**	**Number of Isolates Included**	**Model System**	**Biofilm Quantification Method**	**Antibiotics Tested**
Roveta et al. [48]	3	Microtiter plate	Crystal violet staining	Moxifloxacin
***Acinetobacter baumannii***				
**Reference**	**Number of Isolates Included**	**Model System**	**Biofilm Quantification Method**	**Antibiotics Tested**
Wang et al. [61]	6	Microtiter plate	Crystal violet staining	Meropenem, imipenem, sulbactam, colistin, and tigecycline
***Moraxella catarrhalis***				
**Reference**	**Number of Isolates Included**	**Model System**	**Biofilm Quantification Method**	**Antibiotics Tested**
Roveta et al. [48]	3	Microtiter plate	Crystal violet staining	Moxifloxacin
***Campylobacter jejuni***				
**Reference**	**Number of Isolates Included**	**Model System**	**Biofilm Quantification Method**	**Antibiotics Tested**
Rossi et al. [62]	2	Microtiter plate	Crystal violet staining	Ciprofloxacin, colistin, tetracycline, erythromycin, and meropenem
***Proteus mirabilis***				
**Reference**	**Number of Isolates Included**	**Model System**	**Biofilm Quantification Method**	**Antibiotics Tested**
Li et al. [63]	2	Flow cell system	SYTO 62	Ciprofloxacin
***Helicobacter pylori***				
**Reference**	**Number of Isolates Included**	**Model System**	**Biofilm Quantification Method**	**Antibiotics Tested**
Yonezawa et al. [64]	2	Microtiter plate	Crystal violet staining	Amoxicillin and metronidazole
Wu et al. [22]	9	Microtiter plate	Microscopy and CFU counts	Amoxicillin, clarithromycin, tetracycline, and levofloxacin
***Mycobacteroides abscessus***				
**Reference**	**Number of Isolates Included**	**Model System**	**Biofilm Quantification Method**	**Antibiotics Tested**
Oschmann-Kadenbach et al. [31]	4	Porous glass beads	CFU counts	Amikacin and tigecycline
***Bacillus* spp.**				
**Reference**	**Number of Isolates Included**	**Model System**	**Biofilm Quantification Method**	**Antibiotics Tested**
do Canto Canabarro et al. [65]	4	Microtiter plate	Crystal violet staining	Penicillin, tetracycline, and gentamicin
***Candida* spp.**				
**Authors**	**Number of Isolates Included**	**Model System**	**Biofilm Quantification Method**	**Antibiotics Tested**
Alves et al. [28]	24	Microtiter plate	Crystal violet staining	Fluconazole, voriconazole, anidulafungin, and amphotericin B
Melo et al. [66]	30	Microtiter plate	Crystal violet staining	Amphotericin B and fluconazole
